# Measuring social emergencies through fuzzy qualitative comparative analysis: a calibration framework using normalized general continuous linguistic variables

**DOI:** 10.3389/fsoc.2026.1824793

**Published:** 2026-08-10

**Authors:** Noel Batista Hernandez, Maikel Yelandi Leyva Vazquez, Anabel Mariela Abarca Cruz, Lissette Amelia Alvarado Ajila

**Affiliations:** 1 Universidad Bolivariana del Ecuador, Guayaquil, Ecuador; 2 Centro de Investigación Institucional, Universidad Bernardo O’Higgins, Santiago, Chile

**Keywords:** calibration methods, causal configurations, complexity theory, fuzzy set theory (fsQCA), membership functions, qualitative comparative analysis (QCA), social emergence, social indicators

## Abstract

Social emergencies are complex societal phenomena characterized by nonlinear dynamics, multiple causality, and emergent properties that cannot be reduced to simple causal chains. Understanding the conditions that give rise to such emergencies requires analytical frameworks capable of capturing uncertainty, equifinality, and causal asymmetry. Fuzzy Qualitative Comparative Analysis (fsQCA) offers a set-theoretic approach well-suited to this challenge, combining the interpretive depth of qualitative methods with the rigor of formal logic. However, a central obstacle in its application is the calibration of fuzzy membership functions, which determines how raw social data are translated into degrees of set membership. This paper proposes a novel calibration framework based on the Normalized General Continuous Linguistic Variable (NGCLV), a flexible parametric function capable of taking increasing, decreasing, or convex forms by adjusting a single shape parameter. We introduce one direct calibration method—fitting membership functions to empirical data—and two indirect calibration methods based on partition and linguistic selection approaches. All three methods are demonstrated using a real-world dataset of health indicators across twenty countries, with Life Expectancy as the sociological outcome of interest. We further propose an optimization method that maximizes the joint consistency and coverage indices, automating the identification of the most explanatorily powerful causal configuration. Results show that NGCLVs improve calibration flexibility and semantic interpretability compared to conventional membership functions. The proposed framework advances the methodological toolkit available for sociological research involving small-to-medium case sets, complex causality, and the need to integrate expert knowledge with empirical data.

## 1 Introduction

The concept of emergence refers to phenomena that exhibit novel properties, arising from basic elements but that can be separated from direct causal relationships (Hoel, 2025; Fuentes, 2025). Integrating this idea with general systems theory, Bunge (1977) argues that the central nervous system can be described as a state space, a notion borrowed from physics. From this perspective, consciousness is not simply the sum of the functioning of the brain’s parts, but a property that emerges from the system as a whole, being original and synchronous in human nature.

This principle states that in complex systems, properties emerge that cannot be reduced to or explained by individual units. These new qualities arise from the interactions between components, not from their simple sum. In political science, for example, the formation of public opinion is not reduced to the sum of individual opinions, but rather emerges from processes of communication, influence, and feedback in social networks and media. In sociology, collective behavior in a protest cannot be explained solely by the individual motivations of the participants, but by the group dynamics that emerge in the social context.

All reality includes entropic dynamics, that is, tendencies toward chaos, which can be counteracted by negentropic forces, which act as stabilizing mechanisms. In social systems, this tension is intensified by the influence of human will, which introduces elements that both generate disorder and create order.

Social reality exhibits a plurality of causes that generate diverse manifestations (Byrne and Callaghan, 2022). Direct causes express a relationship between events, in which one triggers the other. In social ecosystems, this relationship is complicated by the presence of causal feedback, as occurs in economics: a fiscal policy can generate a market reaction that, in turn, influences future government decisions.

Multiple causality also occurs. In these cases a condition arises from the interaction of several elements and gives rise to emergent properties that cannot be anticipated by observing each part separately. Recursion refers to operations that act again as a cause within the same system, generating chains of events that reinforce or modify its state.

This dynamic can be expressed as endogeneity, in which the elements themselves apply their internal rules iteratively. As a result, microsystems emerge within a larger unit, which interact at multiple levels and do not respond to linear causal relationships. Through these internal iterations and feedback, behaviors and patterns emerge that cannot be predicted by analyzing their individual parts.

This perspective leads to the consideration that there are areas in which the cause-and-effect relationship becomes indeterminate, making it difficult to establish definitive causal values. In social systems, this uncertainty requires forecasting tools that allow for anticipating scenarios. For example, in urban planning, “gray zones” must be considered where the effects of an intervention cannot be accurately predicted but must be taken into account to make adaptive decisions.

Qualitative Comparative Analysis (QCA) is based on the recognition that reality exists independently of our perception, but that we can only access it through processes of interpretation and reconstruction (Byrne and Callaghan, 2022; Thiem, 2022). This position aligns with complexity theory, which maintains that social phenomena cannot be understood as simple sums of parts, but rather as dynamic systems in which multiple factors interact nonlinearly and generate emergent properties.

To study this complex reality, QCA requires key methodological decisions: selecting the conditions considered relevant to describing and explaining the phenomena, and defining how those conditions are operationalized —that is, how they are translated into observable variables. This process involves recognizing that the researcher not only observes but also constructs the object of study.

QCA offers a dialogic and iterative framework that allows the researcher to move between empirical data (specific cases) and the formulation of causal explanations. This dynamic reflects the principles of complexity theory: multiple causality, feedback, equifinality (multiple paths to the same outcome), and emergence. Thus, QCA articulates a cycle of scientific discovery that conforms to the critical realist approach, which integrates observation, analysis, and theorizing in a continuous process of refinement. Therefore, QCA is an appropriate tool for studying social emergencies.

QCA is the methodology developed by Charles Ragin in the 1980s to study complex social phenomena, especially when working with a small number of cases. Its main objective is to identify causal configurations—that is, combinations of conditions—that explain why a particular outcome occurs (or does not occur) (Ragin, 2009).

Its operation is as follows:

The approach begins with a theory or hypothesis about which conditions might be linked to an outcome.

The cases are coded in a truth table, indicating the presence or absence of each condition.

Boolean logic tools are used to simplify the combinations of conditions that lead to the result.

The analysis allows for the discovery of multiple causal paths (equifinality) and complex relationships such as conjunctural causality (when two conditions must be together for the result to occur).

This tool allows the study of complex causality, where there is no single cause but rather a combination of factors. It is ideal for research with few cases but a wealth of qualitative information, and can work with binary data (presence/absence) or with fuzzy sets (degrees of membership between 0 and 1).

After criticism of crisp QCA, Ragin replaced Boolean values of 0 or 1 with values between 0 and 1 to incorporate uncertainty in measurements, where there are gray areas that cannot be reduced to black and white values (Kumar et al., 2022; Cangialosi, 2023; Farivar, 2023). However, the incorporation of fuzzy sets into the method brought an additional element to consider: how to fuzzify the data. To do this, it is necessary to calibrate the raw data, that is, not only to evaluate the results but also to give them meaning. To illustrate, a country’s Gross Domestic Product can be expressed on a percentage scale (Ragin, 2009). This scale includes negative values (indicating economic decline), positive values (signifying growth), and zero (representing stagnation, with neither growth nor decline). However, an experienced economist does not interpret 1% GDP growth as real economic growth, only 2% or higher, based on existing knowledge.

That is why Ragin, in his Fuzzy QCA or fsQCA, focused on explaining calibration methods. These include a quantitative component, where each element to be measured has an associated truth value, and a substantive or qualitative component, where the result is associated with a nominal value. Another problem is determining the cut-off points where a qualitative change occurs. He proposes the example of water, which can reach negative or positive temperatures; however, the values where the volume of water reaches qualitative changes are 0 °C and 100 °C. In his writings, Ragin proposes a direct calibration method, where experts provide the cut-off points for calibration according to their expertise, and in the indirect method, calibration is performed automatically.

This paper aims to design fsQCA methods that are based on the Normalized General Continuous Linguistic Variables (NGCLV) (González-Caballero et al., 2021). This very recent theory allows for the generation of fuzzy membership functions from three parameters. These parameters can be chosen directly, based on their meanings, or indirectly by fitting them to a data set. They can also be fitted using logical predicates, thus obtaining membership functions that adjust their shapes to the data or the knowledge of experts. This solves a long-standing problem in fuzzy logic, namely, how to select the membership function, which translates to the calibration problem in fsQCA (Singh, 2017; Samanta, 2024; Zhou, 2024). Not using NGCLV implies a specific shape would have to be selected *a priori* (triangular, trapezoidal, Gaussian, sigmoid, among others), which would not always fit the problem under study or the data available. However, once the three parameters of NGCLV are set, it can take approximately any of the aforementioned shapes.

We would also like to mention that QCA has made progress in the neutrosophic field (González-Caballero et al., 2025). A neutrosophic set generalizes the fuzzy set, where, in addition to a membership function, there is also a non-membership function and an indeterminacy function. One approach replaces the classic QCA measures— consistency and coverage, defined by Ragin in a set-theoretic manner—with logical equations, especially those of logical implication.

This paper is structured as follows: a Methods section contains the basic elements of Fuzzy Set theory and the fsQCA model. The Results section details the proposed model, with illustrative examples of its applicability. The following section discusses the results, compares the proposed method with other methods, and provides conclusions. The Appendix provides the computational implementation details and the Julia code.

## 2 Methods

A *fuzzy set A* in a set *X* is defined by a membership function $\mu_{A}:X\to [0,1]$ where for *x* in *X* a truth value of membership to the set *A* is associated with a degree of truthfulness between 0 and 1 (Zimmermann, 2010; Gholamizadeh et al., 2022). Where 0 represents that *x* does not belong to *A*, 1 indicates that *x* fully belongs to *A*, and intermediate values mean partial membership. A value of $\mu_{A}(x)>0.5$ means that *x* is more inside than outside *A*; a value of $\mu_{A}(x)<0.5$ means that *x* is more outside than inside the set *A*. The value 0.5 is indecisive; *x* is as much inside as outside *A*.

For example, if T is the set of tall men, where X = [0, 250] cm height, a function can be defined as shown in Equation 1:


$$\text{sigm}(x;a,c)=\frac{1}{1+e^{-a(x-c)}}$$ (1)

**Figure 1:**
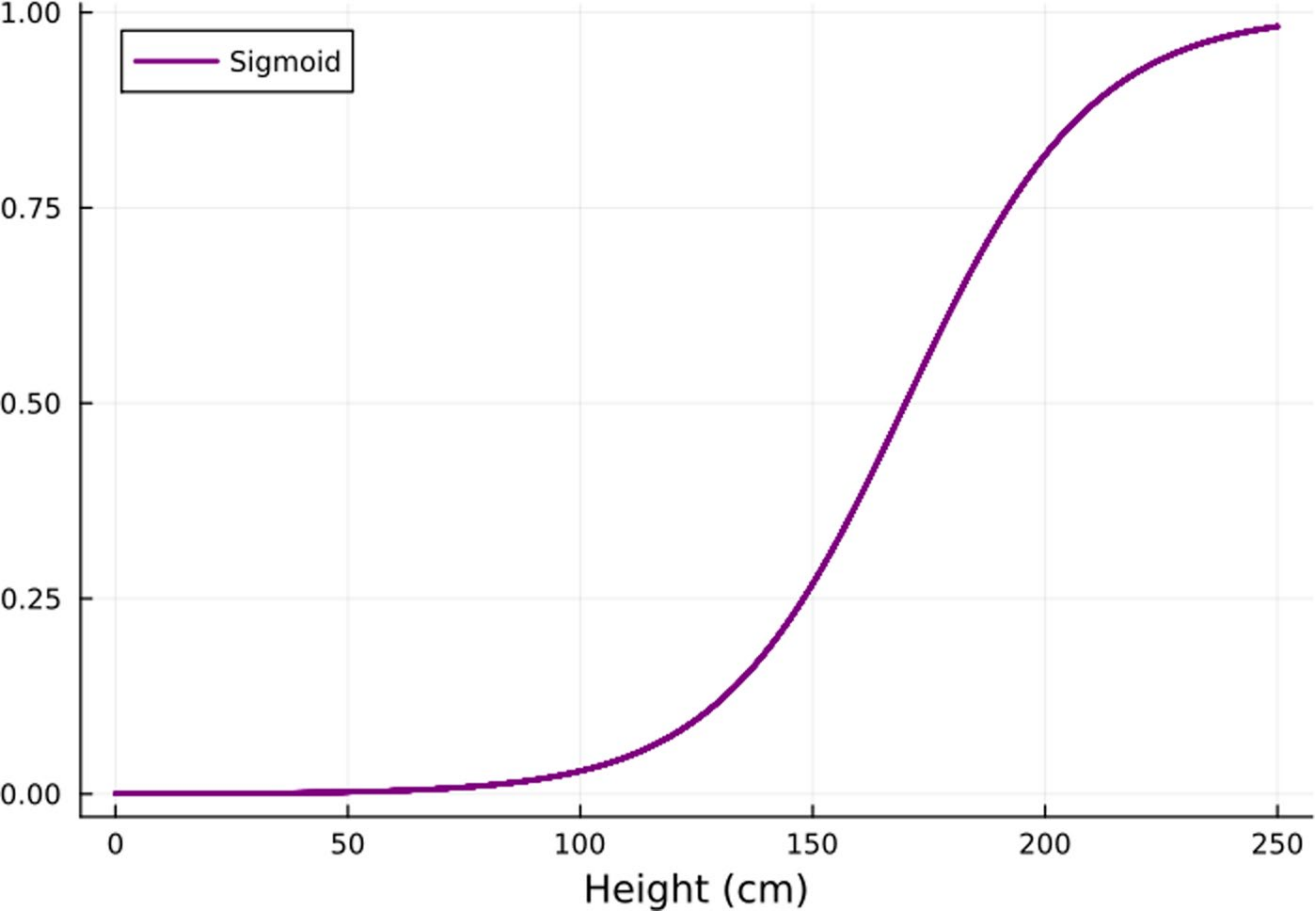
Membership function for the set of “tall men” measured in cm of height.

The result of sigm(x;a=0.05,c=170) representing the set of tall men based on their height measured in cm is graphed in Figure 1.

Figure 1 shows a sigmoid membership function such that sigm(x=170;a=0.05,c=170)=0.5, so the value *c* represents the cutoff point that separates a height considered tall from a height considered short (Singh, 2017; Samanta, 2024; Zhou, 2024). Note that this value is subjective.

**Figure 2:**
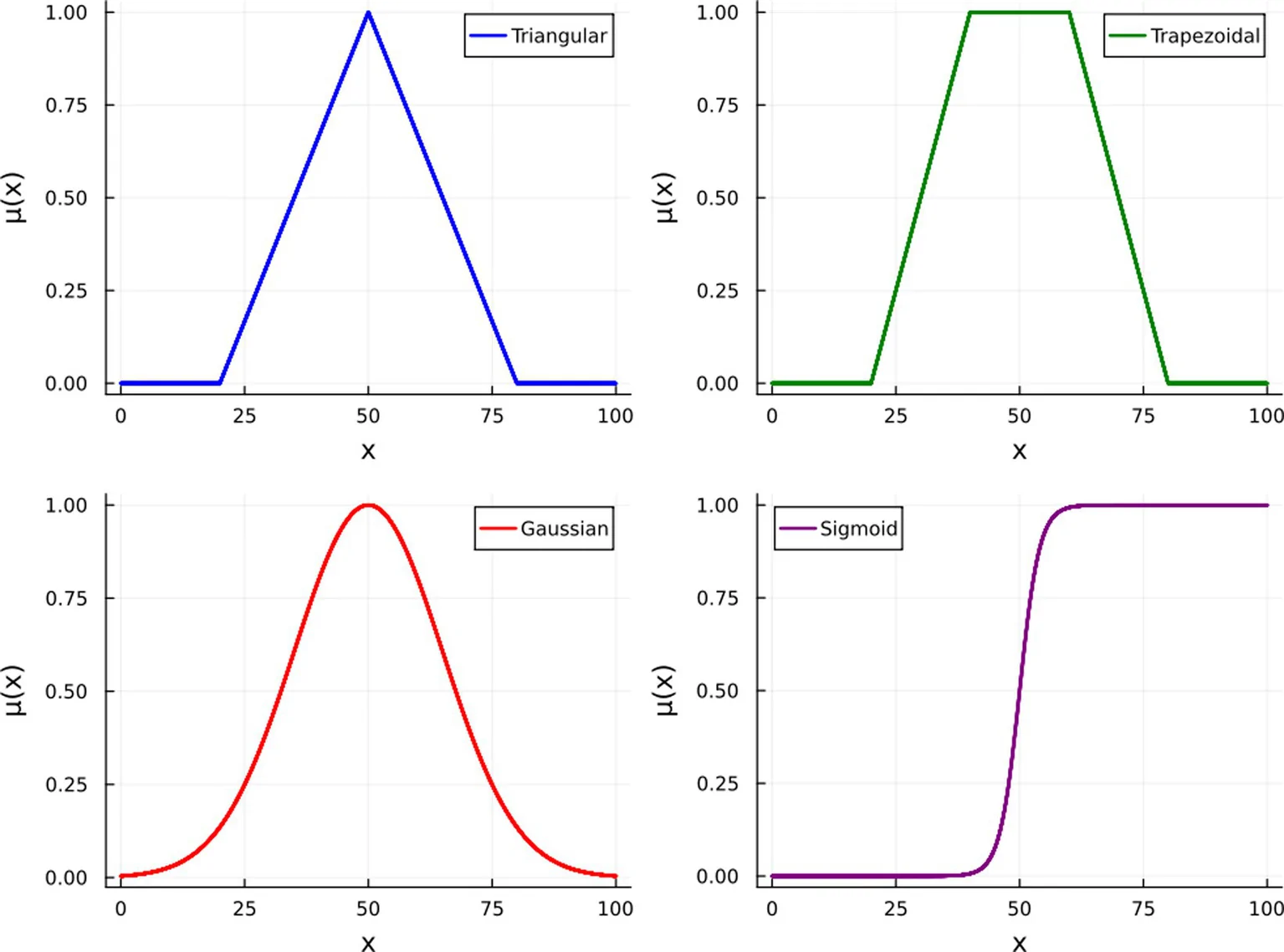
Four forms of the membership function, from top to bottom and from left to right, are triangular, trapezoidal, Gaussian, and sigmoid membership functions.

However, there are other shapes of the membership function, as shown in Figure 2.

A type of *Normalized General Continuous Linguistic* Var*iable* (NGCLV) is defined by the following formula:


$$\mu (x;\alpha ,\gamma ,m\;)=\frac{{\left(\text{sigm}(x;\alpha ,\gamma )\right)}^{m}{\left(1-\text{sigm}(x;\alpha ,\gamma )\right)}^{1-m}}{M}$$ (2)

Where $M=m^{m}{\left(1-m\right)}^{1-m}$ and sigm(x;α,γ) is the membership function of Equation 1 with parameters a=α>0 , c=γ∈ℝ , and m∈[0,1]. In addition, $0^{0}=1$ .

In González-Caballero et al. (2021), it is shown that the formula for M is the maximum of ${\left(\text{sigm}(x;\alpha ,\gamma )\right)}^{m}{\left(1-\text{sigm}(x;\alpha ,\gamma )\right)}^{1-m},$ and therefore Equation 2 has values in the interval [0,1].

The value of m indicates the shape that the membership function will take, unlike the parameters in the membership functions in Figure 2, where changing the parameters results in the same shape of the membership function.

**Figure 3:**
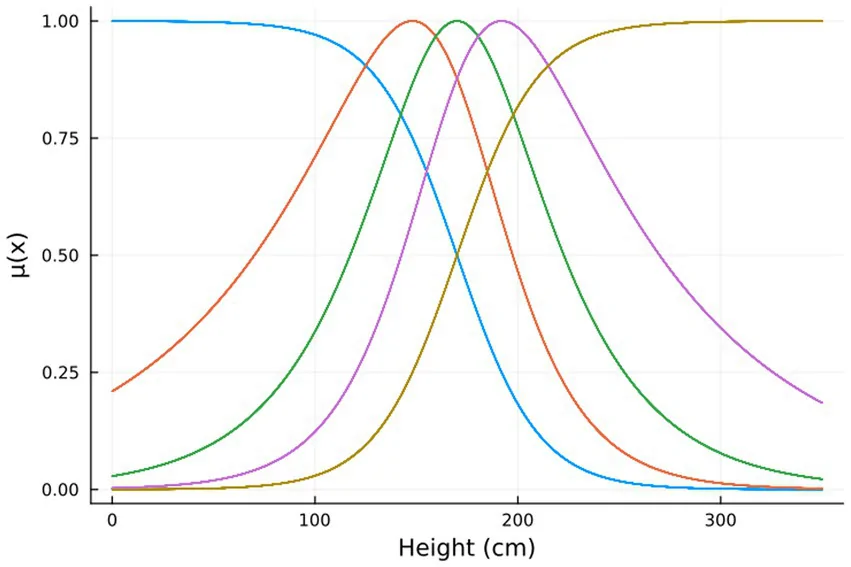
Five forms of the NGCLV by changing the parameter *m* with α=0.05 , γ=170 , and *m* = 0 (blue), 0.25 (red), 0.5 (green), 0.75 (violet), 1 (yellow).

The advantage of NGCLV is that it allows data or knowledge to be adjusted to different forms of the membership function by simply changing a single parameter. Intuitively, in addition to changing the shape by m, α adjusts the precision, and γ influences the crossover point. Note in Figure 3 how by fixing α and γ , and taking various *m* = 0, 0.25, 0.5, 0.75, 1, several shapes were obtained, from a descending sigmoid, an asymmetric one to the right with respect to γ , a symmetric one with respect to γ , an asymmetric one to the left with respect to γ , and an increasing sigmoid.

Zadeh’s fuzzy set theory offers advantages in its application to QCA models. Ragin’s idea for his QCA model is to provide the social sciences with a computational tool based on set-based measurements, where studies can be conducted with several cases intermediate between those typically used in quantitative and qualitative studies.

Ragin criticizes the widespread use of statistical methods in the social sciences. These methods are quantitative and therefore require a “large” number of cases to be effective. A large number is usually defined as a number greater than or equal to 30. Statistical methods do not delve into each case in depth, only into the values of the variables. Correlation is also used as a way to study the behavior of two random variables. The concept of a variable is fundamental to statistics.

He argues that social sciences are naturally expressed verbally, where there are asymmetries in the association between elements, and these are generally nonlinear. However, correlation coefficients in statistics are usually linear and symmetric, which limits the adequate study of the phenomenon. Therefore, he proposes a set-theoretic method on which the QCA method is based.

On the other hand, qualitative methods use a small number of cases—one or perhaps two—where the study is conducted in depth. For example, conducting a qualitative study of a company means delving into the company’s operating mechanisms in great detail. But this does not allow us to understand what happens in other institutions.

Initially, QCA was based on crisp sets, where an element either belonged or did not belong to the set under study. However, Ragin soon received criticism for this, leading him to extend the method to the fuzzy domain with the creation of fsQCA. This led to the problem of calibration.

A fuzzy set is a way to account for the uncertainty that occurs in subjective assessments, as well as capturing the vagueness of natural language. Ragin explains that using fuzzy sets in QCA allows for qualitative results because each fuzzy set has a meaning (e.g., tall man) where each element can be classified as belonging or not belonging. On the other hand, each element is evaluated quantitatively. This is why fsQCA is a method that combines quantitative and qualitative aspects, achieved through the use of fuzzy sets from calibration.

Ragin introduced two calibration methods: the direct and indirect. We explain the first below, and the second later.

In the direct method, three values provided by experts are needed according to current knowledge, viz.: the *threshold for full membership*, the *threshold for full non-membership*, and the *crossover point*. The threshold for full membership is the minimum value that determines that an element is classified according to the label with which the set is provided, on the other hand the threshold for full non-membership is the maximum value that allows us to affirm that the element does not belong to the set, and finally the crossover point is the point of maximum uncertainty for classifying the element as belonging or not belonging to the set. Determining the value of the crossover point is the critical part of this method.

An example he uses is the definition of $5,000 of GDP per capita as the crossover point of the measure of a country’s membership in the group of developed countries. The deviation from this value is a measure of the country’s development. A value below this crossover point indicates that the country does not belong to the group of developed countries; above this value indicates a certain belonging to the group. However, countries with a GDP per capita of $5,000 remain unclassifiable. Specifically, he sets the threshold value at $20,000 for full membership, meaning that a higher value identifies the country as developed. A score of $2,500 or less means the country does not belong to the group of developed countries, so $2,500 is the threshold for full non-membership. For more details (Ragin, 2009), where Ragin uses the log odds as an equivalent but symmetrical scale to determine a country’s classification as developed based on its GDP per capita.

The second method is the indirect method. This is used when there are no clear theoretical criteria for establishing membership thresholds. Instead of defining cutoff points directly, statistical or empirical procedures are used to estimate them. Some procedures are based on empirical data, where data distributions are analyzed to establish membership values. Percentiles or means can be used. It applies to continuous scales, where it is ideal for variables measured on interval or ratio scales.

Among its advantages are that it is more objective when there is no clear theory, it allows working with large volumes of data, and it reduces research bias. However, it can be meaningless if the data are biased or poorly distributed, and it does not replace theoretical judgment when it is available.

Ragin recommends that the researcher organize cases according to different levels of membership, then assign them an initial membership score that is then refined. He proposes the use of fractional methods as part of the logit model, which is available in several statistical packages.

fsQCA method starts from a truth table, with the fuzzy values of the cases of the independent variables $X_{i}$ and for the dependent variables $Y_{i}$ .

Two proposed indices for measuring the relationships between these variables are consistency and coverage. Consistency measures the degree to which a specific condition or combination of conditions produces the same outcome. On the other hand, coverage is a measure of how representative a condition or combination of conditions is.

The consistency and coverage equations proposed by Ragin are shown in Equations 3, 4, respectively (Ragin, 2009):


$$\text{consistency}(X_{i}\leq Y_{i})=\frac{\sum \min(X_{i},Y_{i})}{\sum X_{i}}$$ (3)

$$\text{coverage}(X_{i}\leq Y_{i})=\frac{\sum \min(X_{i},Y_{i})}{\sum Y_{i}}\;\;$$ (4)

Where:

$X_{i}$ is the membership value of case i in the set of causal conditions.

$Y_{i}$ is the membership value of case i in the outcome set.

Consistency and coverage greater than or equal to 0.8 are considered significant.

For example, having a high GDP per capita (X) is assumed to be sufficient for a country to be considered developed (Y). Therefore, the set of developed countries is expected to include as a subset the set of countries with high GDP per capita, and this is an indicator of consistency.

However, another problem remains: even when the consistency of a combination is significant (at least equal to 0.8), this causal combination may result in the same outcome in a small percentage of cases. That is, although a combination of input values can be assured to produce an expected result with high certainty, this combination of inputs may not be the only one or the most frequent; this is why the coverage index complements the consistency index. A coverage index greater than or equal to 0.8 indicates that the combination of inputs is significant in obtaining an outcome, and any other combination will be infrequent. Following the previous example, the set of countries with high GDP per capita is expected to cover the set of developed countries.

## 3 Results

In this section, we offer three approaches to calibration using NGCLVs. These are a direct calibration method and two indirect calibration methods. Of the latter two, the first uses the algorithm to obtain the interpretation with linguistic labels through the data as it appears in González-Caballero et al. (2021), that is, given the data, the membership functions associated with the linguistic values are automatically generated, then one of these linguistic values is chosen and taken as a reference in each variable, to associate a fuzzy truth value with each raw value.

The second indirect method allows for the automatic acquisition of membership functions such that the values of consistency and coverage are maximized. This method is obtained by optimizing truth predicates, as shown in González-Caballero et al. (2021). In other words, truth values are automatically sought based on the data, such that coverage and consistency are maximal. Each of these methods is explained below.

### 3.1 Direct calibration method using NGCLV

The sigmoid function can be considered to depend on a parameter *β*, where the truth value equal to 0.1 is reached, this is called the “almost inadmissible value.” It means that β is the maximum threshold where the truth values can be interpreted as having little belonging or what Ragin called “mostly but not fully out of the target set.”

So, having that *γ* is the crossover point or “acceptable value,” which is where the sigmoid membership function reaches a truth value of 0.5, experts can take advantage of these properties to construct an NGCLV membership function according to their criteria for selecting these two points.

That is, the direct method consists of the expert determining two values, one is *β* (for almost false), one is γ (for indeterminate), and the parameter *α* is obtained by Equation 5.


$$\alpha =\frac{\ln(0.9)-\ln(0.1)}{\gamma -\beta }$$ (5)

Where *ln(x)* is the natural logarithm.

We recommend that the parameter *m* be chosen as *m* = 0 to indicate the label “Low,” “Little,” and so on; *m* = 1 indicates the linguistic value “High,” “A lot,” and so on; while *m* = 0.5 means “Neither much nor little,” “More or less,” “Medium,” “Neither high nor low,” and so on. In this last membership function, *γ* is where the truth value of 1 is obtained, and truth values of 0.5 are reached in $x_{1,2}=\gamma \pm \frac{1}{\alpha }\text{arccosh}(7)$ . Although other values for the parameters *m* can be used, they will not necessarily remain shapes that are acceptable to the expert. For example, for *m* = 0.25, there are no direct formulas to calculate the most important parameters; in this case, the truth value of 1 is not reached for either *β* or γ. However, taking these observations into account, the use of these parameters is not entirely ruled out.

Example 1. Suppose we wish to study the behavior of health indicators in 20 countries with diverse economic development and political and health systems. See Table 1 for more details.

**Table 1:** Raw data for six health indicators from twenty countries.

Country	#Fertility rate	#Infant mortality	#Maternal mortality ratio	#Physicians per thousand	#Life expectancy
Australia	1.74	3.1	6	3.68	82.7
Brazil	1.73	12.8	60	2.15	75.7
Canada	1.5	4.3	10	2.61	81.9
Chile	1.65	6.2	13	2.59	80
Costa Rica	1.75	7.6	27	2.89	80.1
Cuba	1.62	3.7	36	8.42	78.7
Dominican Republic	2.35	24.1	95	1.56	73.9
Finland	1.41	1.4	3	3.81	81.7
Germany	1.56	3.1	7	4.25	80.9
India	2.22	29.9	145	0.86	69.4
Japan	1.42	1.8	5	2.41	84.2
Mexico	2.13	11	33	2.38	75
Morocco	2.42	19.2	70	0.73	76.5
Nepal	1.92	26.7	186	0.75	70.5
Nigeria	5.39	75.7	917	0.38	54.3
Poland	1.46	3.8	2	2.38	77.6
Saudi Arabia	2.32	6	17	2.61	75
South Africa	2.41	28.5	119	0.91	63.9
South Korea	0.98	2.7	11	2.36	82.6
Sweden	1.76	2.2	4	3.98	82.5

Source: Gigasheet (n.d.).

For the present analysis, we used a subset of the Global Country Information Dataset 2023 [compiled by Nidula Elgiriyewithana (Gigasheet, n.d.)]. From the full dataset, which contains 195 countries and a wide range of socioeconomic indicators, we extracted a sample of 20 countries based on data availability and relevance to our research question. The selected variables focus exclusively on health indicators, including life expectancy, infant mortality rate, maternal mortality rate, fertility rate, and physicians per 1,000 population. This subset was used to calibrate fuzzy membership functions and conduct the subsequent fsQCA analysis.

The experts are asked what value or values they consider to be almost insignificant for each of the indicators to define *β*, and also which value or values they consider acceptable (the crossover point) to define *γ*. The results are summarized in Table 2. When dealing with estimates where the desired index is average, then γ would be the maximum, and there would be two βs as crossover points. When *m* = 0, the membership function 0.9 is obtained for β.

**Table 2:** Threshold values for designing the NGCLV.

Parameter/Indicator	#Fertility rate	#Infant mortality	#Maternal mortality ratio	#Physicians per thousand	#Life expectancy
β	1.3 and 3	5	5	1	50
γ	2.1	25	70	3	70
α	Estimated at ~3.1427	0.109861	0.033804	1.098612	0.109861
m	Estimated at ~0.5737	0	0	1	1

**Figure 4:**
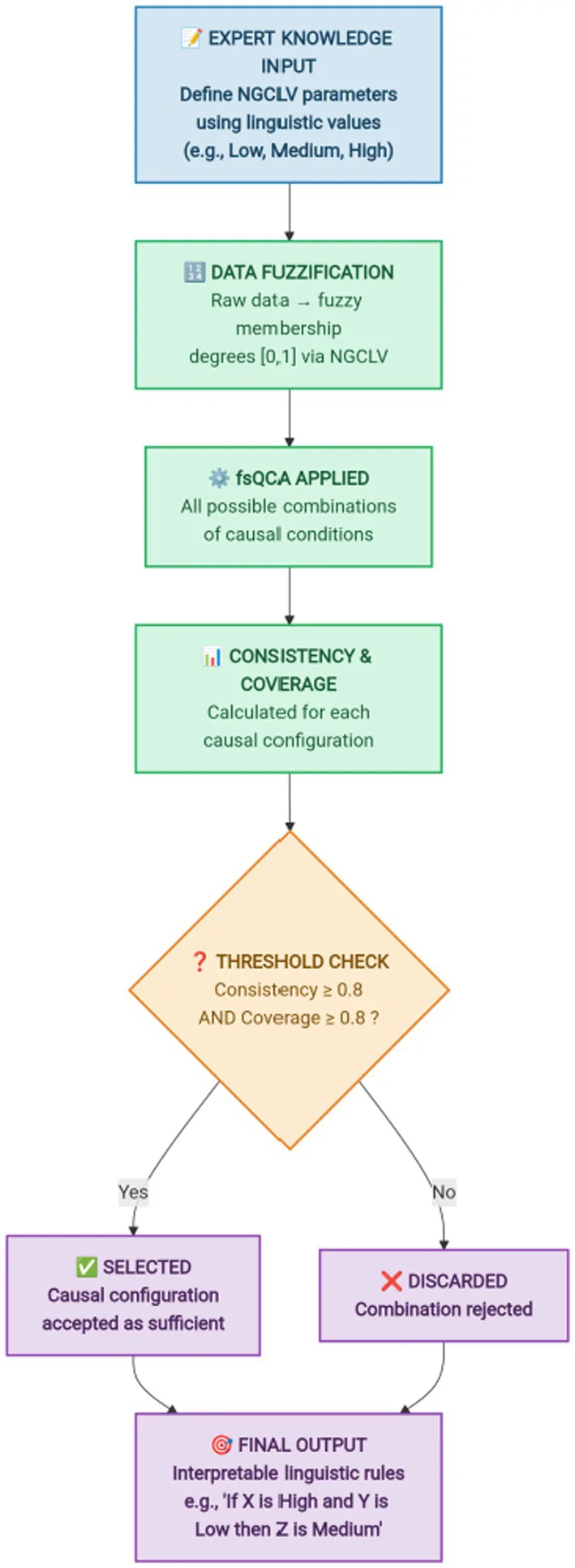
Flowchart of direct calibration based on expert knowledge. This figure illustrates the workflow for direct calibration, where the user defines NGCLV parameters using linguistic values, followed by data fuzzification, fsQCA evaluation, and selection of configurations with consistency and coverage ≥ 0.8, producing interpretable linguistic rules.

The results in Table 2 indicate that the parameters “# Infant Mortality” and “#Maternal Mortality Ratio” are calibrated to the “Low” label because they are assigned the value *m* = 0. “# Physicians per thousand” and “# Life Expectancy” are calibrated as “High,” so *m* = 1 is taken. In the index “# Fertility Rate,” we want to obtain a parameter *m* that satisfies that the NGCLV is 0.5 in 1.3 and 3. On the other hand, it reaches the value 1 in 2.1. This is why a specific *m* cannot be chosen, and it is necessary to estimate using an optimization method. This shows the flexibility of NGCLVs, which can adapt to different shapes; however, in some cases, this may not have a solution. See Figure 4 with the obtained functions. Note the different types of shapes of the membership function.

Table 3 contains the values from Table 1, fuzzified with the NGCLV obtained with the parameters from Table 2.

**Table 3:** Fuzzified data of five health indicators from twenty countries, using NGCLVs with the parameters in Table 2.

Country	#Fertility rate	#Infant mortality	#Maternal mortality ratio	#Physicians per thousand	#Life expectancy
Australia	0.782	0.917	0.897	0.679	0.801
Brazil	0.773	0.793	0.584	0.282	0.652
Canada	0.582	0.907	0.884	0.394	0.787
Chile	0.707	0.887	0.873	0.389	0.75
Costa Rica	0.79	0.871	0.811	0.47	0.752
Cuba	0.682	0.912	0.759	0.997	0.722
Dominican Republic	0.972	0.525	0.3	0.171	0.606
Finland	0.512	0.93	0.906	0.709	0.783
Germany	0.632	0.917	0.894	0.798	0.768
India	0.999	0.369	0.073	0.087	0.484
Japan	0.519	0.927	0.9	0.343	0.826
Mexico	0.995	0.823	0.777	0.336	0.634
Morocco	0.943	0.654	0.5	0.076	0.671
Nepal	0.912	0.453	0.019	0.078	0.514
Nigeria	0.024	0.004	0	0.053	0.151
Poland	0.55	0.911	0.909	0.336	0.697
Saudi Arabia	0.982	0.89	0.857	0.394	0.634
South Africa	0.948	0.405	0.16	0.091	0.338
South Korea	0.255	0.921	0.88	0.331	0.8
Sweden	0.798	0.924	0.903	0.746	0.798

Let us take the variable “Life Expectancy” as output and the variables “Fertility Rate,” “Infant Mortality,” “Maternal Mortality Ratio,” and “Physicians per thousand” as the independent variables. Let us denote them by V1, V2, V3, and V4. We can ask: if a country has a suitable fertility rate, low infant mortality, low maternal mortality ratio, and a high number of physicians per thousand people, will it consequently exhibit a high life expectancy?

The results of the calculations are shown in Table 4.

**Table 4:** fsQCA results obtained from direct calibration using NGCLV.

Terms	Consistency	Coverage	Combined
v_1_*v_2_*v_3_*v_4_	1	0.661756	0.809406
v_1_*v_2_*v_3_	0.959917	0.734736	0.852871
v_1_*v_2_*v_4_	1	0.666388	0.812234
v_1_*v_3_*v_4_	1	0.661756	0.809406
v_2_*v_3_*v_4_	0.990941	0.556577	0.742301
v_1_*v_2_	0.942674	0.83794	0.906191
v_1_*v_3_	0.959917	0.734736	0.852871
v_1_*v_4_	1	0.667907	0.813159
v_2_*v_3_	0.88375	0.864824	0.901629
v_2_*v_4_	0.971151	0.562424	0.746190
v_3_*v_4_	0.990941	0.556577	0.742301
v_1_	0.789023	0.860267	0.813883
v_2_	0.853213	0.968029	0.933395
v_3_	0.88375	0.864824	0.901629
v_4_	0.960696	0.566145	0.748655

The indices calculated with the fsQCA software result in the causal configurations with consistency and coverage greater than 0.8, as follows (Ragin and Davey, 2016):

Fertility rate and infant mortality
Infant mortality and maternal mortality ratio
Maternal mortality ratio

### 3.2 Calibration method with indirect linguistic selection using NGCLV

González-Caballero et al. (2021) propose an algorithm, where given the measured quantitative data and the number of linguistic values to be modeled, they assign each linguistic value an automatically generated fuzzy membership function. Roughly speaking, the algorithm is as follows:

Input:

Measured data scaled between 0 and 100. Equation 6 is used to scale them to 0–100.


$$x_{\text{rescaled}}=(\frac{x_{\text{original}}-x_{\min}}{x_{\max}-x_{\min}})100$$ (6)

Precision h value, which is the value greater than 0 and less than 100, such that it determines the length of the 0.5-cut interval.

$\hat{n}$ : Number of linguistic values to be obtained.

From h, the number of elements in the initial partition is calculated using Equation 7:


$$n=\text{floor}(\frac{100}{h})+1$$ (7)

Where *floor*(x) is the function that approximates x to the smallest integer.

Now for i = 2, …, n-1 the NGCLVs with the parameters m = 0.5 are used, $\alpha =\frac{5.267831587699267}{h}$ for all NGCLVs, and $\gamma_{i}=0,h,2h,\ldots ,100$ .

That is, we have the set of functions:


$$\mu_{i}(x;\frac{5.267831587699267}{h},(i-1)h,0.5)$$

Specifically, for i = 1, we have the sigmoid:

$\mu_{1}(x;\frac{5.267831587699267}{h},\frac{h}{2},0.0)$ , and for i = n, we have another sigmoid:


$$\mu_{1}(x;\frac{5.267831587699267}{h},100-\frac{h}{2},1.0)$$

These last two functions are a modification of the original algorithm, which allows for more adequate interpretability of the results.

For each pair of consecutive NGCLV functions in the partition, a merge is performed. Then, we will have n-1 partitions from the merge of two consecutive membership functions. In each partition, two consecutive functions are merged, and the others are left unchanged.

A merge is obtained from two membership functions that intersect at a point within the partition. From these, a new membership function is formed with the minimum 0.5-cut of one of the functions and the maximum 0.5-cut of the other function. The maximum of the new function is calculated using the data as a weighted average with weights depending on the data values evaluated in both original functions. In this way, the maximum of the new function will be reached in the part of the interval corresponding to the original function with the largest data values.

Within all partitions, a distance function is calculated between all elements in a given partition. The partition with the largest distance is then selected.

If the *n* obtained is equal to the $\hat{n}$ desired, we encountered the desired partition.

Otherwise, we apply the procedure in step 3 to the last partition obtained, which will have one less element in the partition, because two functions were merged.

Once the membership functions for each variable are obtained, the linguistic value to be used for calibration is selected for each variable. The data in the original table are then fuzzified using the membership function for each variable.

The fsQCA method is applied to the data in the fuzzified table.

To better understand this calibration method, let us revisit Example 1 and apply the method to the elements in Table 1.

Example 2: The original data are those we used in Table 1. Let us take h = 10 as the precision, and set $\hat{n}=3$ , to indicate the linguistic values “High,” “Medium,” and “Low.” Applying Equation 7, we get several elements in the initial partition equal to 11.

**Figure 5:**
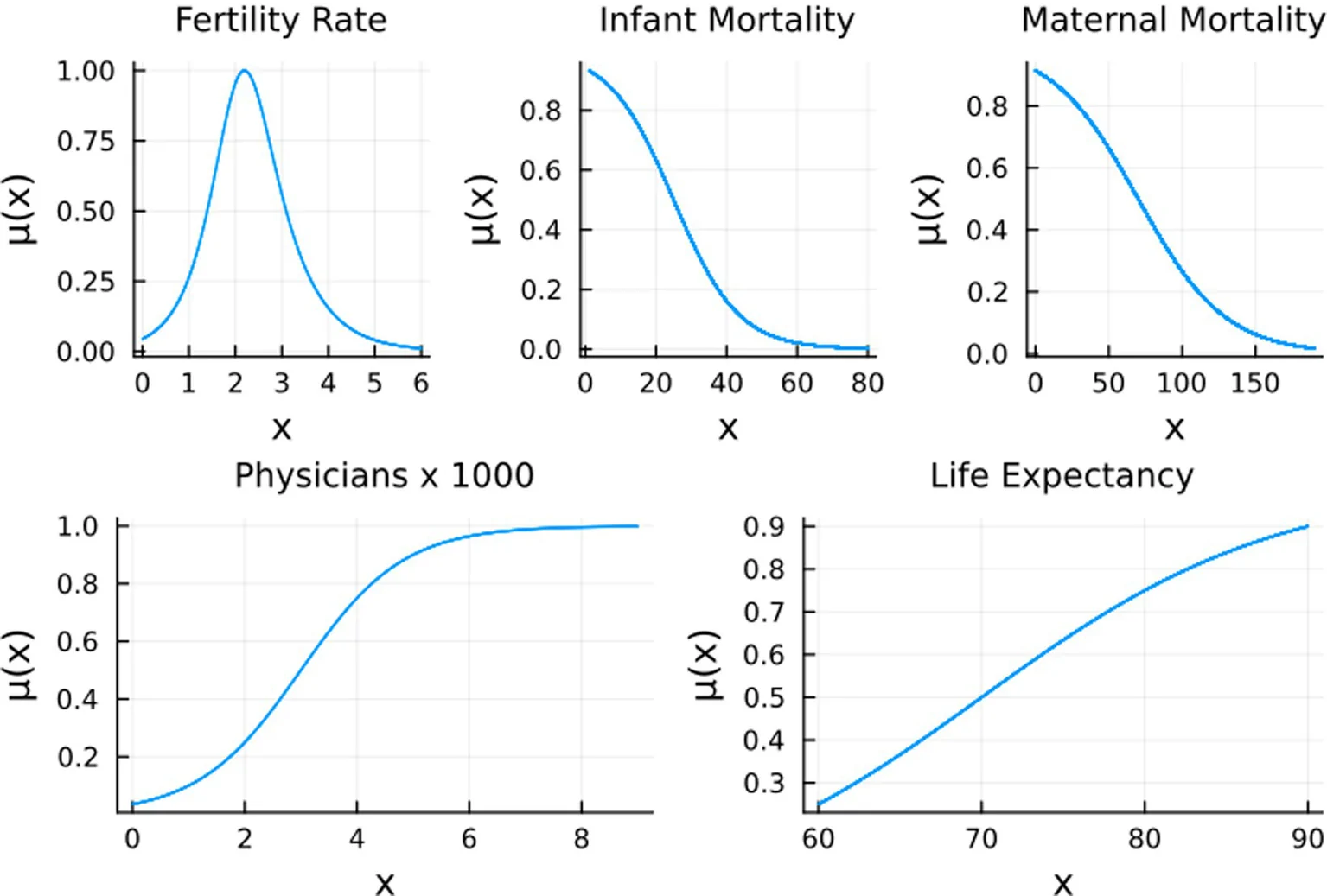
Plot of the NGCLVs membership functions with the parameters obtained from Table 2.

**Figure 6:**
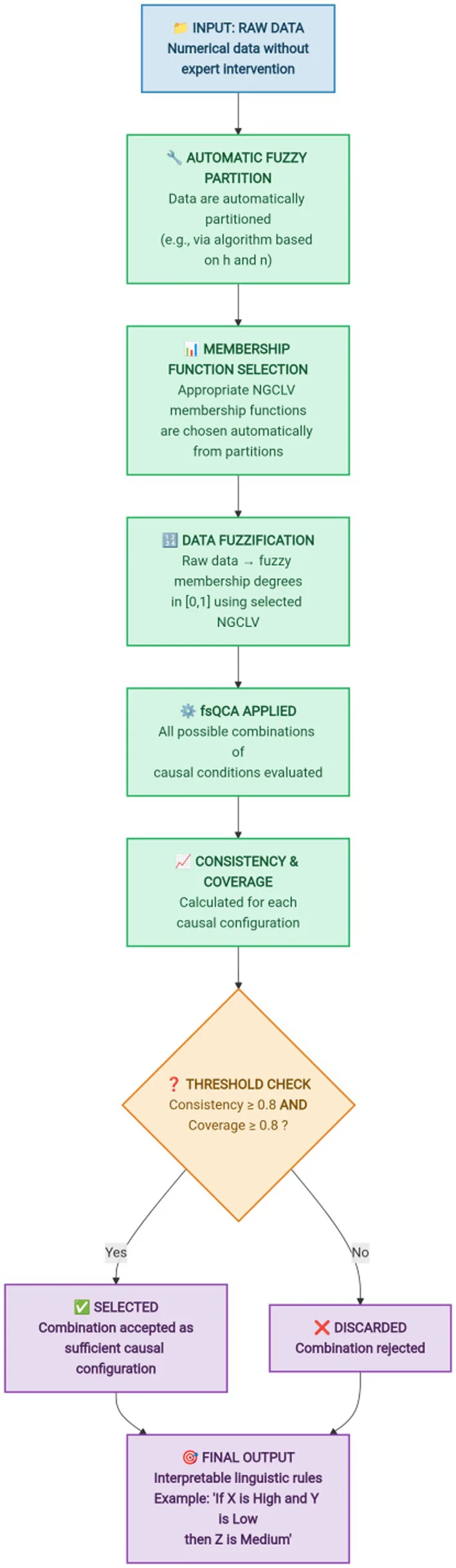
Flowchart of automatic indirect calibration (no expert intervention). This figure shows the automatic calibration process: raw data are loaded, an automatic fuzzy partition is performed, membership functions are selected, data are fuzzified, fsQCA is applied, and configurations with both consistency and coverage ≥ 0.8 are selected, yielding linguistic rules.

Recalling that the initial data for each variable are scaled between 0 and 100 using Equation 6, we will have the partition shown in Figure 5, where all the NGCLVs functions have parameters *m* = 0.5 except the first and the last (i.e., they are symmetrical with respect to *γ*), all alphas are equal to *α* = 1.0535663175398535, while *γ_i_* = (i-1)5, for i from 0 to 21. The two extreme ones are sigmoid (see Figure 6).

**Figure 7:**
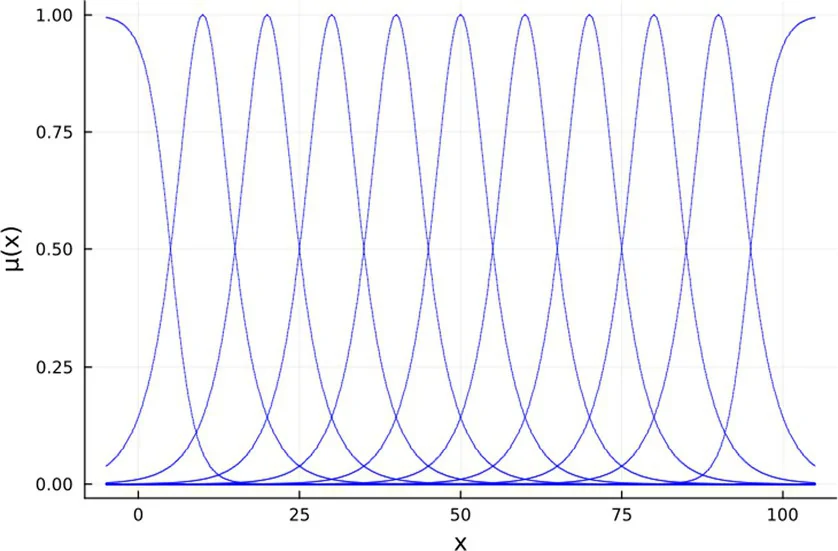
Initial partition for data scaled between 0 and 100, with h = 10. The length of each 0.5-cut is equal to 10.

**Figure 8:**
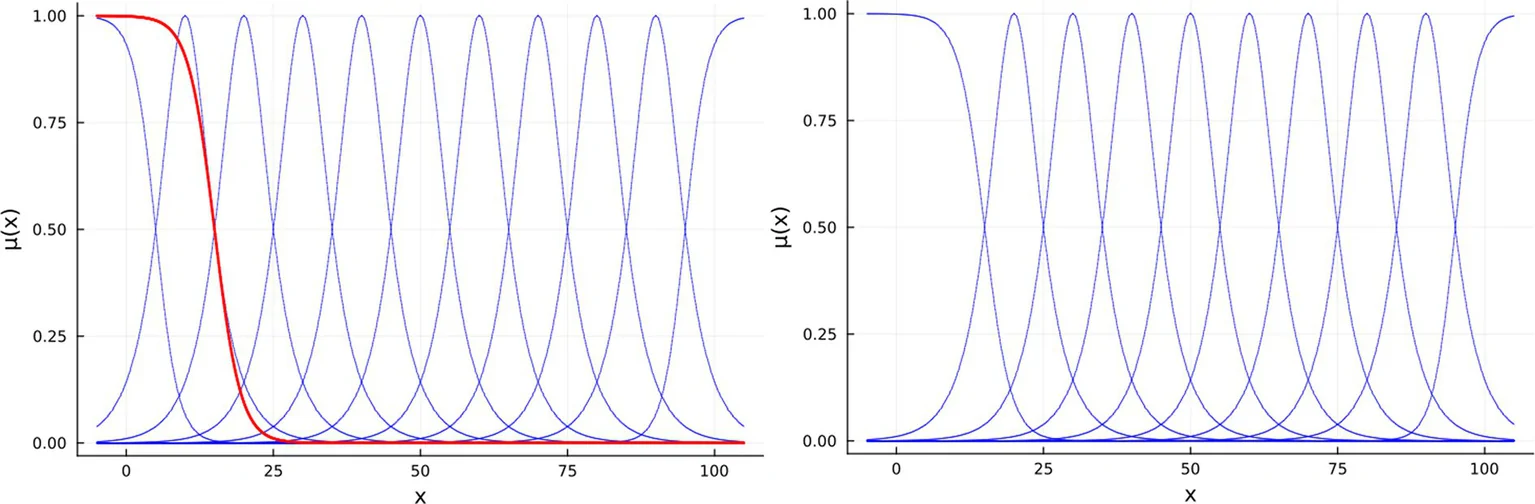
New partition obtained by merging the first two membership functions. On the left, the red line shows the merged function. On the right, the new partition.

After applying the merging of the first two membership functions in Figure 7, which are $\mu_{1}(x;\alpha =0.526783,\gamma_{1}=5,m=0),$ and $\mu_{2}(x;\alpha =0.526783,\gamma_{2}=10,m=0.5),$ a new membership function is obtained $\mu_{1,2}(x;\alpha =0.4517411672214805,\gamma =15.0,m=0).$ . The new partition consists of this new function and all the other functions in the original partition. See Figure 8, which shows a partition with the functions from Figure 5 and the first two merged, in red. The new partition appears on the right.

When the membership functions are merged $\mu_{2}(x;\alpha =0.526783,\gamma_{2}=10,m=0.5\;)$ with $\mu_{3}(x;\alpha =0.526783,\gamma_{3}=20,m=0.5),$ a new function is obtained which, together with the other original membership functions, forms a second partition, and so on until 10 partitions of the space made up of 10 membership functions are obtained.

The selection of the partition that goes to the next phase is the one such that the distance between all its components is the greatest among the set of partitions.

Since we still have a partition with 10 functions and want to get to one with only 3, the same process is repeated for this new partition. That is, 9 partitions are obtained from the one in the previous step, where two consecutive membership functions are merged.

This is repeated until a partition of only three functions is obtained. It is also repeated for each of the variables.

**Figure 9:**
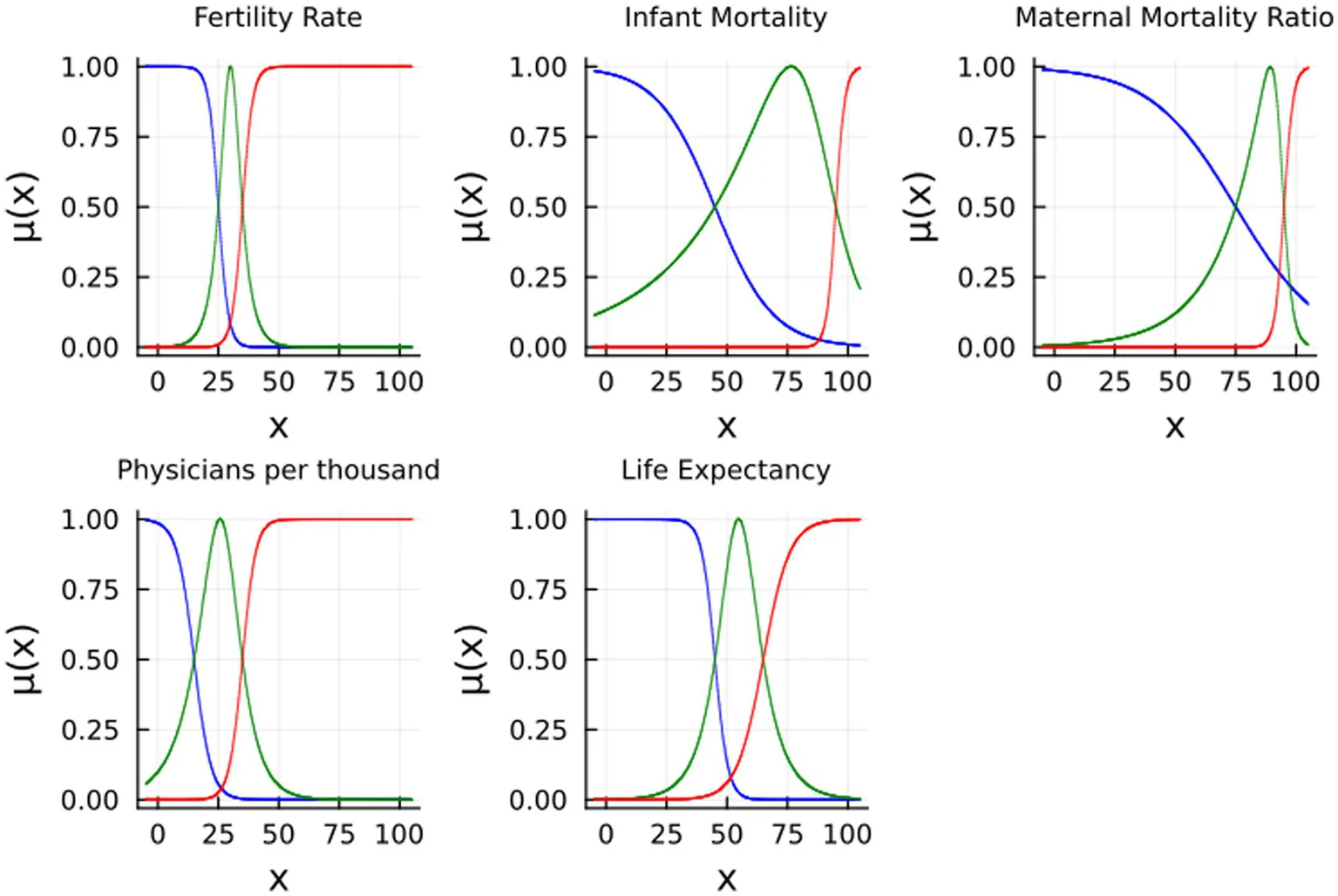
Partitions obtained for each variable. The membership functions are represented in blue for Low, in green for Medium, and in red for High. The data are on a scale between 0 and 100.

**Figure 10:**
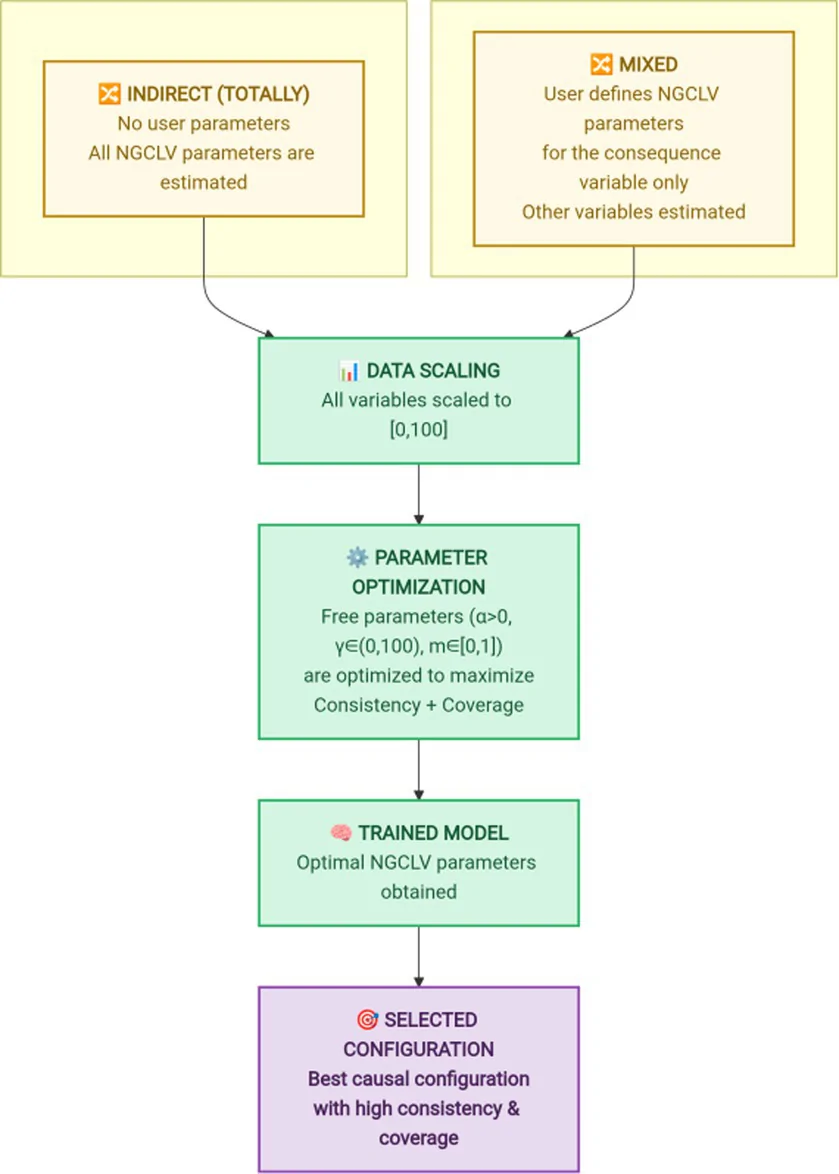
Flowchart of hybrid calibration: totally indirect vs. mixed variant. This figure presents two variants: totally indirect (no user parameters) and mixed (user defines only the outcome NGCLV parameters). After scaling data to [0,100], free parameters are optimized to maximize Consistency + Coverage. Configuration with max (Consistency + Coverage) is selected.

The results of the calculations were as follows, as shown in Table 5 and see Figures 9, 10.

**Table 5:** Results of obtaining the NGCLVs for each of the linguistic values of each of the linguistic variables.

Variable	Parameters (α, γ, m) for each linguistic value
#Fertility rate	Low: (0.483, 25.053, 0.0)
	Medium: (0.527, 30.0, 0.5)
	High: (0.463, 35.000, 1.0)
#Infant mortality	Low: (0.084, 45.000, 0.0)
	Medium: (0.136, 85.942, 0.218)
	High: (0.527, 95.0, 1.0)
#Maternal mortality ratio	Low: (0.057, 75.000, 0.0)
	Medium: (0.492, 93.472, 0.116)
	High: (0.527, 95.0, 1.0)
#Physicians per thousand	Low: (0.283, 15.000, 0.0)
	Medium: (0.269, 27.072, 0.410)
	High: (0.365, 35.000, 1.0)
#Life expectancy	Low: (0.368, 45.000, 0.0)
	Medium: (0.264, 54.412, 0.525)
	High: (0.182, 65.000, 1.0)

### 3.3 From the partitions of each variable, we select the membership function of interest for calibration

As in the previous example, we have:

Infant Mortality and Maternal Mortality Ratio are calibrated as “Low”; Physicians per thousand and Life Expectancy are calibrated as “High”; Fertility Rate is calibrated as “Medium.” Fuzzifying the data shown in Table 1 with the membership function corresponding to the linguistic value selected for calibration yields the results shown in Table 6.

**Table 6:** Fuzzified data of five health indicators from twenty countries, using the NGCLVs calibrated with the indirect method.

Country	Average #Fertility rate	#Infant Mortality low	#Maternal mortality ratio low	#Physicians per thousand high	#Life expectancy high
Australia	0.06920894	0.9726649	0.98554955	0.90091688	0.99578351
Brazil	0.06520519	0.92273524	0.97993021	0.00864632	0.7681236
Canada	0.0165244	0.96883518	0.98519271	0.06583027	0.99315178
Chile	0.04046293	0.961689	0.9849194	0.06045933	0.97851683
Costa Rica	0.07345727	0.95544189	0.98357685	0.20088147	0.9797615
Cuba	0.03382945	0.9708109	0.98265242	1	0.95375115
Dominican Republic	0.96185379	0.76999022	0.97519638	0.00059773	0.52512235
Finland	0.00965377	0.97731728	0.98581161	0.9425636	0.99227077
Germany	0.0236443	0.9726649	0.98546114	0.99180858	0.98747413
India	0.88856591	0.63539884	0.96650198	2.4885E-05	0.06646171
Japan	0.01024789	0.9762975	0.98563743	0.02762566	0.99830578
Mexico	0.63170077	0.93599804	0.98296611	0.02419166	0.68375191
Morocco	0.797286	0.85318029	0.97867616	1.3788E-05	0.84361538
Nepal	0.20096764	0.71414847	0.95723981	1.5099E-05	0.12218903
Nigeria	0	0.00995696	0.19532975	2.8125E-06	7.1642E-06
Poland	0.01301314	0.97049026	0.98589792	0.02419166	0.91339985
Saudi Arabia	0.99486746	0.9625098	0.98454724	0.06583027	0.68375191
South Africa	0.82559398	0.67106879	0.97133828	3.1229E-05	0.00248542
South Korea	0.00074019	0.97383679	0.98510216	0.02213742	0.99551969
Sweden	0.07796489	0.97523304	0.98572478	0.97261732	0.99523944

Table 7 shows the results obtained from applying the fsQCA algorithm to the data in Table 6.

**Table 7:** fsQCA results obtained from calibration with indirect linguistic selection using NGCLV.

Terms	Consistency	Coverage	Combined
v_1_*v_2_*v_3_*v_4_	1	0.03233	0.178905
v_1_*v_2_*v_3_	0.639434	0.225362	0.237362
v_1_*v_2_*v_4_	1	0.03233	0.178905
v_1_*v_3_*v_4_	1	0.03233	0.178905
v_2_*v_3_*v_4_	0.996757	0.362118	0.598746
v_1_*v_2_	0.639434	0.225362	0.237362
v_1_*v_3_	0.57	0.225362	0.150121
v_1_*v_4_	1	0.03233	0.178905
v_2_*v_3_	0.83361	0.987427	0.926856
v_2_*v_4_	0.996757	0.362118	0.598746
v_3_*v_4_	0.994531	0.363002	0.599476
v_1_	0.568975	0.225362	0.150121
v_2_	0.83361	0.987427	0.926856
v_3_	0.76588	0.995907	0.834946
v_4_	0.990471	0.363141	0.599591

Now, calculating with the fsQCA software, the causal configurations with consistency and coverage that exceed the threshold of 0.8 are:

Infant mortality and maternal mortality ratio,
Infant mortality.

### 3.4 Calibration method with indirect linguistic selection using NGCLV, selecting the optimal causal configuration

The third calibration method can be purely indirect or a combination of methods. However, we believe the combination method yields more useful results.

Let X1, X2,…, Xn be the causal variables and Y be the output variable. Each of them has an associated NGCLV with membership functions $\mu_{1}(x_{1};\alpha_{1},\gamma_{1},m_{1}),$ $\mu_{2}(x_{2};\alpha_{2},\gamma_{2},m_{2}\;),$ …, $\mu_{n}(x_{n};\alpha_{n},\gamma_{n},m_{n}\;),$ for the causal variables; and $\mu_{n+1}(y;\alpha_{n+1},\gamma_{n+1},m_{n+1}\;),$

Thus, we have two variants,

Variant #1 (Pure direct method),

The optimization problem is solved,

maxconsistency+coverage of X1, X2,…, Xn, Y.

For $\alpha_{1},\alpha_{2},\ldots ,\alpha_{n+1}>0;$ $\gamma_{1},\gamma_{2},\ldots ,\gamma_{n+1}\in (0,100)$ ; $m_{1},m_{2},\ldots ,m_{n+1}\in [0,1]$ as parameters to be estimated. That is, the parameters of all NGCLVs that maximize the sum of consistency and coverage must be sought.

Variant #2 (Indirect method):

The decision-maker sets the parameters of the NGCLV of Y, $\alpha_{n+1}=\hat{\alpha },\gamma_{n+1}=\hat{\gamma },m_{n+1}=\hat{m},$ obtaining ${\hat{\mu }}_{n+1}(y;\hat{\alpha },\hat{\gamma },\hat{m}),$ , and with this fuzzification of the outcome, the following problem is solved:

maxconsistency+coverage of X1, X2,…, Xn; Y.

$\alpha_{1},\alpha_{2},\ldots ,\alpha_{n}>0;$ $\gamma_{1},\gamma_{2},\ldots ,\gamma_{n}\in (0,100);$ $m_{1},m_{2},\ldots ,m_{n}\in [0,1]$ are the parameters to be estimated. ${\hat{\mu }}_{n+1}(y;\hat{\alpha },\hat{\gamma },\hat{m})$ remain fixed.

Example 3. Suppose we continue with the analysis of the data in Table 1. Again, Life Expectancy is fixed as an outcome. We wish to know under what causal configuration a high Life Expectancy is obtained; therefore, the fuzzified values of Life Expectancy are taken by the direct method that we show in Table 3, last column. The problem now consists of maximizing consistency+coverage for the other three variables. Using the metaheuristic method Evolutionary Centers Algorithm (ECA) coded in Julia language, we obtained the following two results in Table 8 as the most useful:

**Table 8:** Results of obtaining NGCLVs that optimize consistency+coverage with outcome life high expectancy for the causal configuration v_1_*v_2_*v_3_.

Variable	Parameters (*α*, *γ*, *m*) for each linguistic value
#Fertility rate	*α_1_* = 0.039669295574999666
	*γ_1_* = 0.010000000002088262
	*m_1_* = 0.3194592599367439
#Infant mortality	*α_2_* = 0.0302132088031504
	*γ_2_* = 18.133282139629305
	*m_2_* = 0.07717350810279747
#Maternal mortality ratio	*α_3_* = 0.15522199032056053
	*γ_3_* = 0.010000000231598364
	*m_3_* = 0.8365366983926281

In the results of Table 8 the restrictions $\alpha_{1},\alpha_{2},\alpha_{3}\in [0.01,3],$ $\gamma_{1},\gamma_{2},\gamma_{3}\in [0.01,99.99]$ and $m_{1},m_{2},m_{3}\in [0,1]$ were set.

The consistency obtained is 0.9707215585716245, and the coverage is 0.9793602415342565.

v_1_*v_2_*v_3_*v_4_ configuration was calculated, v_4_ gave unusable results, which is why we decided to study it separately. The calculation of the parameters was as follows:

*α_4_* = 0.10000000000000003, *γ_4_* = 99.98999999999998, and *m_4_* = 0.09566177215211133.

The restrictions imposed were $\alpha_{4}\in [0.1,3],$ $\;\gamma_{4}\in [0.01,99.99],$ and $m_{4}\in [0,1].$

The consistency is 0.934667410422837, and the coverage is 0.9459832017212838.

**Figure 11:**
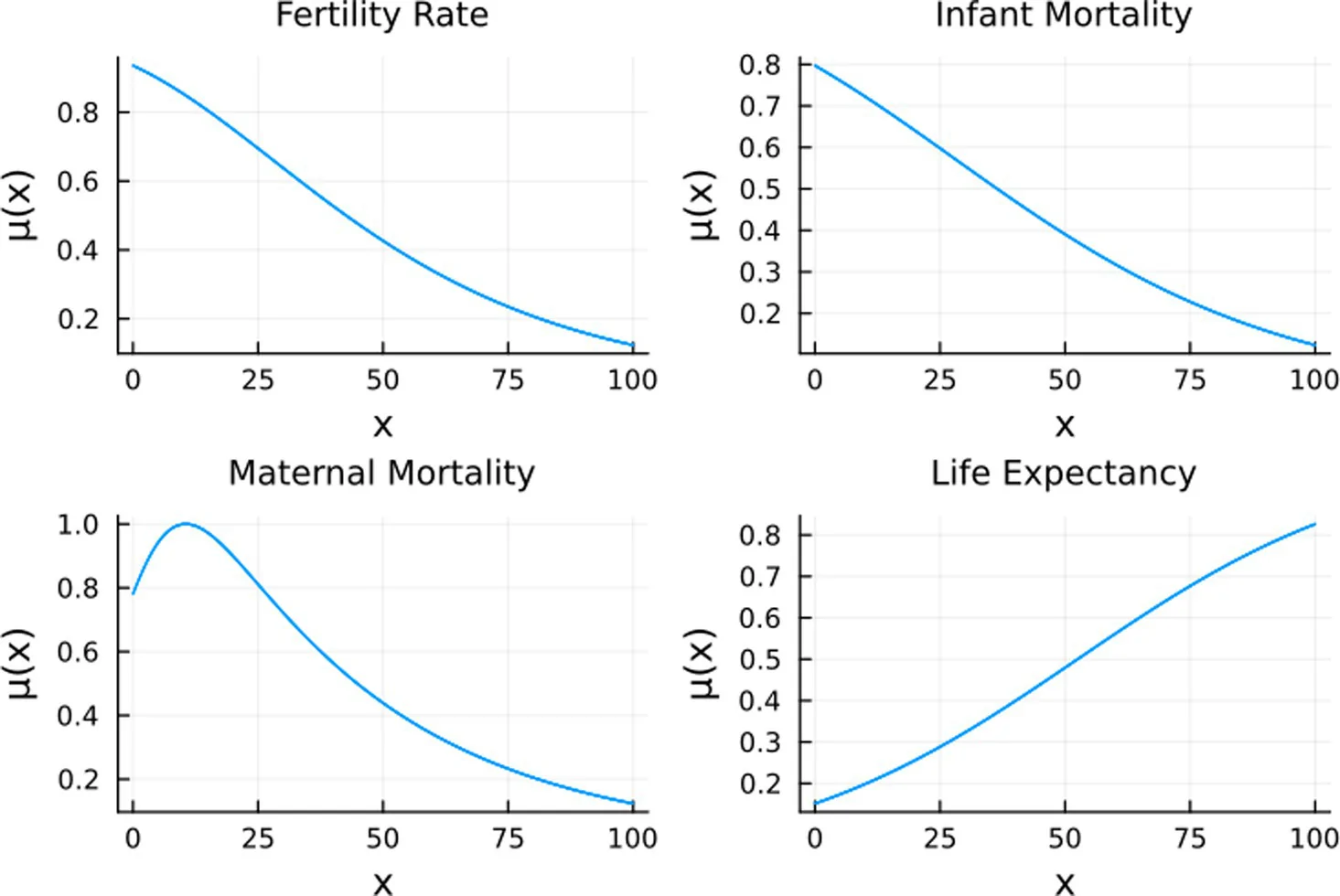
Results of applying variant 2 of example 3. The membership functions of the causal configuration v_1_*v_2_*v_3_ and v_5_ are plotted.

Figure 11 contains the results of the fuzzification of the causal configuration and the outcome for v_1_*v_2_*v_3_ and v_4_, respectively. Let us recall that the plot is based on a data scale between 0 and 100.

**Figure 12:**
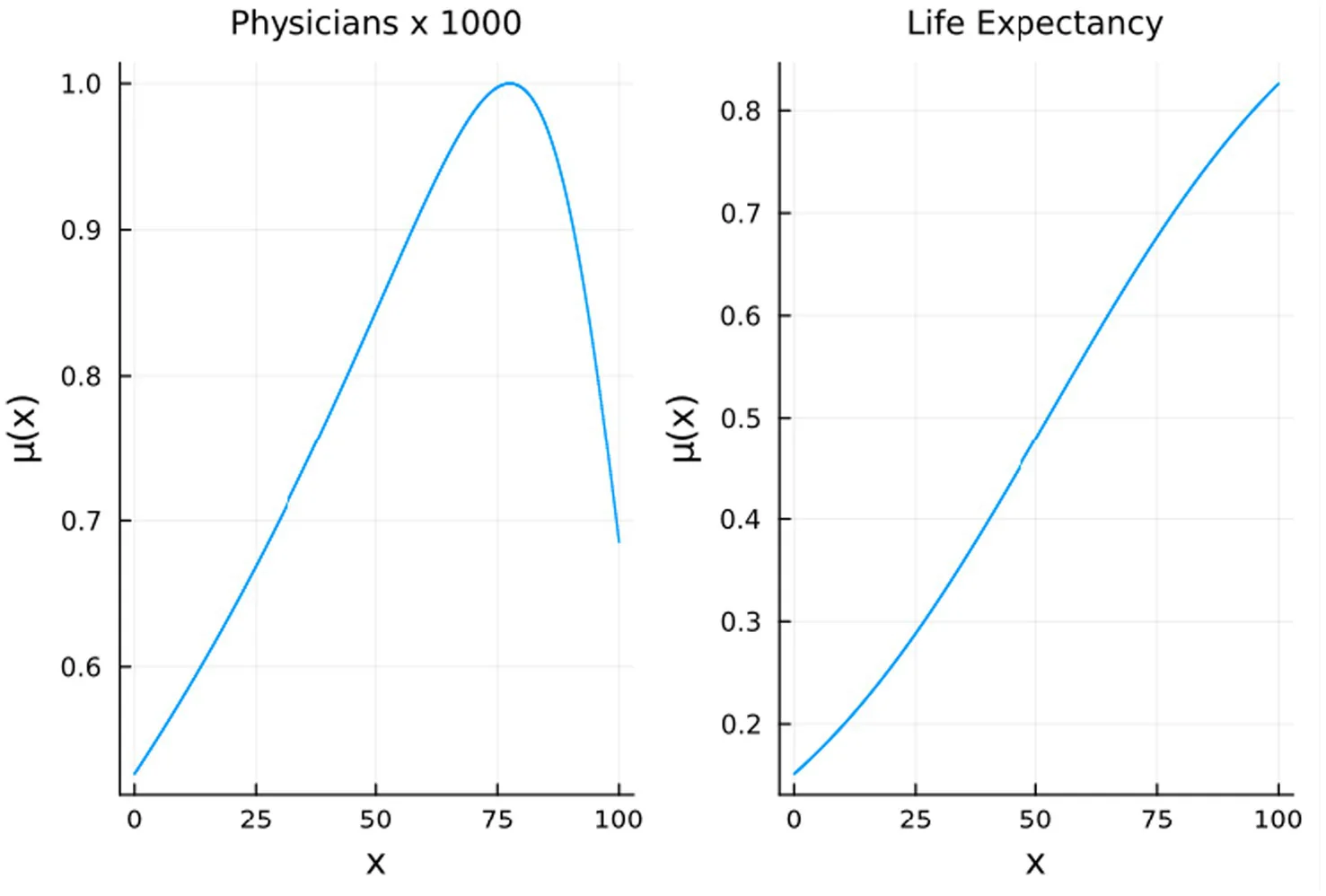
Results of applying variant 2 of example 3. The membership functions of the causal configuration v_4_ and v_5_ are plotted.

Figure 12 graphs the causal configuration of v_4_ and v_5_.

The results in Figures 11, 12 show that the combination of a low fertility rate, low infant mortality, and an average maternal mortality results in high life expectancy. Also, when the number of physicians per 1,000 inhabitants is around a high value, there will be a high life expectancy.

The surprising matter is that maternal mortality and physicians for 1,000 have convex membership functions, which need to be analyzed.

Table 9 shows the results obtained with the help of the fsQCA software, based on the indirect calibration method proposed by Ragin:

**Table 9:** fsQCA results obtained from indirect calibration using Ragin’s method.

Terms	Consistency	Coverage	Combined
V1	1	0.022592	0.149552
V1*V2	1	0.022585	0.149531
V1*V2*V3	1	0.022585	0.149531
V1*V2*V3*V4	1	0.018178	0.134151
V1* V2*V4	1	0.018178	0.134151
V1*V3	1	0.022592	0.149552
V1*V3*V4	1	0.018185	0.134175
V1*V4	1	0.018185	0.134175
V2	1	0.02567	0.159414
V2*V3	1	0.02567	0.159414
V2*V3*V4	1	0.02121	0.144907
V2*V4	1	0.02121	0.144907
V3	1	0.026193	0.161033
V3*V4	1	0.021734	0.146686
V4	1	0.021734	0.146686

Ragin also proposed a direct method, which can be used from the software with the help of the `calibrate (x, n1, n2, n3)` function, where n1 corresponds to the full non-membership threshold (0.05), n2 is the cross-over threshold (0.5), and n3 is the full membership threshold (0.95).

Returning to Table 9, maximum consistency is observed in all causal configurations, but coverage is less than 0.05. It should be noted that Ragin uses percentiles as parametric thresholds to define his membership functions, both in the direct and indirect methods.

With Ragin’s method, it is not possible to determine the state of the variable; however, the proposed calibration method allows conclusions of the type:

If “Infant Mortality” is “Low” and “Maternal Mortality” is “Low” Then “Life Expectancy” is “High,” which is easily understandable for the researcher.

## 4 Limitations

The methods proposed in this article have some limitations. One of them is the limitation inherent to the QCA and FsQCA method: one must have a not-small number of cases to carry out the study. Recall that due to the number of cases, this method is not as exhaustive as qualitative methods, which are appropriate for one or two case studies at most.

On the other hand, a sample size as large as in statistical methods is not required.

Another limitation is that the supervision of a specialist is required to ensure that there is sufficient variety of data, with representativeness of possible cases.

Specifically, the proposed methods are not easily understandable for any sociologist wishing to conduct a study. First, they would need mathematical knowledge of the model and then programming skills to perform the calculations, which are complex. Therefore, it is recommended that they work for now with the help of a specialist who has the skills described above. However, it is promising that in the future the calculations could be performed in a user-friendly way, allowing the sociologist to perform the calculations and obtain the desired results transparently.

Finally, we must note that a mathematical model like this should only serve as information and support for the sociology specialist. A mathematical model should never be interpreted by an inexperienced person, as it should serve only as support.

## 5 Discussion

This paper introduces a previously undefined way of calibrating fsQCAs. The use of the General Continuous Linguistic Variables allows for the automatic acquisition—through data or expert determination—of direct or indirect calibration of fuzzy causal configurations. This solves two fundamental problems. The first one is that it is possible to perform indirect and automatic calibration with fsQCA. The original method has the difficulty that, in addition to having to determine the membership functions for calibration, it can also be very exhaustive when analyzing all possible combinations of causal configurations, which becomes very cumbersome for a relatively large number of variables. However, with the method we propose, the desired configurations are obtained, always with a semantic meaning.

The second problem is in obtaining membership functions in fuzzy logic. The General Continuous Linguistic Variables are a viable alternative, as they can take different forms and adapt to the available data. This is not possible with other types of membership functions, which have a predetermined shape. This article has shown that this “flexibility” allows for greater approximation in the results to represent the real problem to be studied, and also in a fairly simple manner.

The nonlinearity of social emergencies causes inexplicable and sudden phenomena to emerge within society. fsQCA, as defined by Ragin, is a way to study these phenomena, since it considers multiple causality, feedback, equifinality, and emergence among its basic principles. Fuzziness allows for the capture of uncertainty. However, as explained above, the methods proposed in this paper offer new possibilities for the development of this method.

As future work, we propose applying the methods to more existing problems. In this way, their usefulness will be demonstrated in practice. Furthermore, we can explore other indices that have appeared in recent literature to calculate the fsQCA. Specifically, the consistency and coverage indices based on neutrosophic logic are of interest, as they can be adapted to fuzzy logic (González-Caballero et al., 2025).

## 6 Conclusion

This paper has presented a novel calibration framework for Fuzzy Qualitative Comparative Analysis (fsQCA) grounded in the General Normalized Continuous Linguistic Variable (NGCLV). The framework addresses a longstanding methodological challenge in the sociological application of fsQCA: the translation of raw social data into semantically meaningful fuzzy membership values. By offering three calibration approaches—direct fitting, partition-based indirect calibration, and linguistically guided indirect calibration—the proposed methods provide researchers with flexible tools that can accommodate both data-driven and expert-informed perspectives.

The empirical application to cross-national health indicators demonstrates that the NGCLV framework yields consistent and interpretable results, with consistency and coverage values meeting or exceeding the 0.8 threshold recommended in the fsQCA literature. The optimization approach introduced in Example 3 enables automatic identification of the causal configuration that maximizes both indices simultaneously, reducing researcher discretion in a step that has traditionally been subject to ambiguity.

From a sociological standpoint, the relevance of this contribution lies in its alignment with complexity theory’s core premises: that social outcomes emerge from nonlinear interactions among multiple conditions, and that analytical tools must be capable of capturing such complexity. fsQCA, as extended by the methods proposed here, constitutes a robust bridge between qualitative social theorizing and quantitative formal analysis.

Future research should apply these calibration methods to a broader range of sociological case studies, including longitudinal analyses and comparative regional studies. The integration of neutrosophic consistency and coverage indices also represents a promising avenue for extending the framework to contexts of radical uncertainty.

## Data Availability

The original contributions presented in the study are included in the article/Supplementary material, further inquiries can be directed to the corresponding author.
