# Supplementary material for: Measuring social emergencies through fuzzy qualitative comparative analysis: a calibration framework using normalized general continuous linguistic variables
Source: Front Sociol. 2026 Aug 10;11:1824793. doi: 10.3389/fsoc.2026.1824793 (PMC13500326; doi:10.3389/fsoc.2026.1824793)
Supplement: Supplementary file 1 [file Supplementary_file_1.docx]

**Supplementary Material**

*Measuring social emergencies through fuzzy qualitative comparative analysis: a calibration framework using normalized general continuous linguistic variables*

Frontiers in Sociology — Manuscript ID 1824793

**Appendix**

This appendix provides the code and computational details for the calibration methods described in

the paper. All calculations were implemented in the Julia programming language.

**A. NGCLV Membership Function**

The following function computes the Normalized General Continuous Linguistic Variable (NGCLV)

membership value:

function gclv(x,A,C,m)

if(m!=0 && m!=1)

M = (m^m)*((1-m)^(1-m));

else

M = 1;

end

sfx = sf(x,A,C)

return (sfx^m)*((1-sfx)^(1-m))/M;

end

function sf(x,A,C)

return (1 + exp(-A*(x - C))).^(-1)

end

**B. Direct Calibration Method**

The direct method uses expert-defined parameters to calibrate the membership functions. The data

from Table 1 are defined as follows:

Fertility_Rate = [1.74 1.73 1.5 1.65 1.75 1.62 2.35 1.41 1.56 2.22 1.42 2.13 2.42 1.92 5.39 1.46 2.32

2.41 0.98 1.76]

Infant_Mortality = [3.1 12.8 4.3 6.2 7.6 3.7 24.1 1.4 3.1 29.9 1.8 11 19.2 26.7 75.7 3.8 6 28.5 2.7

2.2]

Maternal_Mortality = [6 60 10 13 27 36 95 3 7 145 5 33 70 186 917 2 17 119 11 4]

Physiciansx1000 = [3.68 2.15 2.61 2.59 2.89 8.42 1.56 3.81 4.25 0.86 2.41 2.38 0.73 0.75 0.38 2.38

2.61 0.91 2.36 3.98]

Life_Expectancy = [82.7 75.7 81.9 80 80.1 78.7 73.9 81.7 80.9 69.4 84.2 75 76.5 70.5 54.3 77.6 75

63.9 82.6 82.5]

Fuzzification for the direct method is performed as follows (using the expert-defined parameters from

Table 2):

FRF = [gclv(xi,3.1427,2.1, 0.5737) for xi in Fertility_Rate]

IMF = [gclv(xi, 0.109861.25, 0) for xi in Infant_Mortality]

MMF = [gclv(xi, 0.033804.70, 0) for xi in Infant_Mortality]

P1000F = [gclv(xi, 1.098612,3, 1) for xi in Physiciansx1000]

LEF = [gclv(xi, 0.109861.70, 1) for xi in Life_Expectancy]

The fuzzified values (Table 3) are then processed using the fsQCA software to obtain the causal

configurations reported in Table 4.

**C. Indirect Calibration Method (Linguistic Selection)**

The indirect method uses an automatic fuzzy partitioning algorithm, adapted from

González‑Caballero et al.10. The procedure is as follows:

Step 1: Define the target number of linguistic terms, 𝑛̂, and the initial 0.5-cut length, h (with 0 < h <

100).

Step 2: Scale the data to [0, 100] and set the domain 𝐷= [−

ℎ

2 , 100 +

ℎ

2].

Step 3: Create the initial fuzzy partition:

𝐹𝑃𝑗= {𝜇1

𝑗, 𝜇2

𝑗, ⋯, 𝜇𝑛

𝑗} with 𝑛= ⌊

ℎ⌋+ 1 and for each i = 1,…, n:

𝜇𝑖

𝑗(𝑥) = 𝑁𝐺𝐶𝐿𝑉(𝑥; 𝛼𝑖(ℎ), 𝛾𝑖(ℎ), 0.5) if 𝑖≠1, 𝑛;

𝜇1

𝑗(𝑥) = 𝑁𝐺𝐶𝐿𝑉(𝑥; 𝛼1(ℎ), 𝛾1(ℎ), 0), and 𝜇𝑛

𝑗(𝑥) = 𝑁𝐺𝐶𝐿𝑉(𝑥; 𝛼𝑛(ℎ), 𝛾𝑛(ℎ), 1).

For 𝛼𝑖(ℎ) =

ℎ𝑎𝑟𝑐𝑐𝑜𝑠ℎ(7) and 𝛾𝑖(ℎ) = (𝑖−1)ℎ.

Step 4: If 𝑛= 𝑛̂, stop. Otherwise, proceed to Step 5.

Step 5: For each possible merge of two consecutive membership functions, 𝜇𝑘

𝑗(𝑥) and 𝜇𝑘+1

𝑗

(𝑥),

compute the following:

1. Weights:

𝜔𝑚

𝑗= ∑

𝜇𝑚

𝑗(𝑥𝑞

𝑗)

𝑞=1,2,⋯,𝑁

, 𝑚 = 𝑘, 𝑘+ 1.

2. Weighted core:

• 𝑦𝑘

𝑗= 𝑐𝑜𝑟𝑒(𝜇𝑘

𝑗), 𝑦𝑘+1

𝑗

= 𝑐𝑜𝑟𝑒(𝜇𝑘+1

𝑗

), and 𝑦̂𝑘

𝑗=

𝜔𝑘

𝑗𝑦𝑘

𝑗+𝜔𝑘+1

𝑗

𝑦𝑘+1

𝑗

𝜔𝑘

𝑗+𝜔𝑘+1

𝑗

.

3. 0.5‑cut endpoints:

• 𝑧1

𝑗= 𝑖𝑛𝑓{𝑥∈𝐷|𝜇𝑘

𝑗(𝑥) ≥0.5},

𝑧2

𝑗= 𝑖𝑛𝑓{𝑥∈𝐷|𝜇𝑘+1

𝑗

(𝑥) ≥0.5}.

4. Merged function:

• The merged membership function is the NGCLV that interpolates the three points

(𝑦̂𝑘

𝑗, 1), (𝑧1

𝑗, 0.5), and (𝑧2

𝑗, 0.5).

Step 6: For each candidate partition (obtained by merging one pair), compute the global distance:

```
𝐷𝑛=
𝑛(𝑛−1) ∑
𝑑(𝜇𝑘, 𝜇𝑙)
𝑘,𝑙=1,2,⋯,𝑛;𝑘≠𝑙
 with 𝑑(𝜇𝑘, 𝜇𝑙) =
𝑁∑
|𝜇𝑘(𝑥𝑞) −𝜇𝑙(𝑥𝑞)|
𝑞=1,2,⋯,𝑁
```

.

Select the partition that maximizes 𝐷𝑛, i.e., 𝑀𝑛= 𝑎𝑟𝑔𝑚𝑎𝑥𝑖(𝐷𝑛

𝑖).

Step 7: Update the partition by merging the pair identified in Step 6, set n→n-1, and return to Step 4.

The Julia function partition(x, n, h) implements the above algorithm.

Applying this algorithm to the data of the direct method example yields the following results (see

also Table 5):

partition(Fertility_Rate ,3,10) (With 𝑛̂ = 3, h = 10):

0.483 25.053 0.0

0.527 30.0 0.5

0.463 35.000 1.0

Infant_Mortality:

0.084 45.000 0.0

0.136 85.942 0.218

0.527 95.0 1.0

Maternal_Mortality:

0.057 75.000 0.0

0.492 93.472 0.116

0.527 95.0 1.0

Physiciansx1000:

0.283 15.000 0.0

0.269 27.072 0.410

0.365 35.000 1.0

Life_Expectancy:

0.368 45.000 0.0

0.264 54.412 0.525

0.182 65.000 1.0

Each row corresponds to the parameters 𝛼, 𝛾, 𝑚 for the linguistic labels "Low" (first row), "Medium"

(second row), and "High" (third row).

For example, to evaluate the rule:

If Fertility_Rate is Medium AND Infant_Mortality is Low AND Maternal_Mortality is Low AND

Physicians_per_1000 is High THEN Life_Expectancy is High, we select the corresponding

parameters:

FRF = [gclv(xi, 0.527, 30.0, 0.5) for xi in Fertility_Rate]

IMF = [gclv(xi, 0.084, 45.000, 0.0) for xi in Infant_Mortality]

MMF = [gclv(xi, 0.057, 75.000, 0.0) for xi in Infant_Mortality]

P1000F = [gclv(xi, 0.365, 35.000, 1.0) for xi in Physiciansx1000]

LEF = [gclv(xi, 0.182, 65,000, 1.0) for xi in Life_Expectancy]

The resulting fuzzified vectors are then processed with the fsQCA software to obtain the results

shown in Table 7.

**D. Hybrid Calibration (Optimization of Consistency + Coverage)**

In Example 3, the parameters of the consequence variable (Life_Expectancy) are fixed using the

direct method: α = 0.109861, γ = 70, and m = 1.0. With these fixed values, the parameters of the

remaining NGCLVs are estimated by maximizing the sum of consistency and coverage.

The following Julia code performs this optimization:

```
using Metaheuristics, Statistics

# Data (Table 1, 20 countries)
Fertility_Rate = [1.74, 1.73, 1.5, 1.65, 1.75, 1.62, 2.35, 1.41, 1.56, 2.22,
                  1.42, 2.13, 2.42, 1.92, 5.39, 1.46, 2.32, 2.41, 0.98, 1.76]

Infant_Mortality = [3.1, 12.8, 4.3, 6.2, 7.6, 3.7, 24.1, 1.4, 3.1, 29.9,
                    1.8, 11, 19.2, 26.7, 75.7, 3.8, 6, 28.5, 2.7, 2.2]
Maternal_Mortality = [6, 60, 10, 13, 27, 36, 95, 3, 7, 145,
                      5, 33, 70, 186, 917, 2, 17, 119, 11, 4]
Physicians_per_1000 = [3.68, 2.15, 2.61, 2.59, 2.89, 8.42, 1.56, 3.81, 4.25, 0.86,
                       2.41, 2.38, 0.73, 0.75, 0.38, 2.38, 2.61, 0.91, 2.36, 3.98]
Life_Expectancy = [82.7, 75.7, 81.9, 80, 80.1, 78.7, 73.9, 81.7, 80.9, 69.4,
                   84.2, 75, 76.5, 70.5, 54.3, 77.6, 75, 63.9, 82.6, 82.5]

# Scale variables to [0,100]
function scale_to_100(data)
    minv = minimum(data)
    maxv = maximum(data)
    return 100 .* (data .- minv) ./ (maxv - minv)
end

fertility_s   = scale_to_100(Fertility_Rate)
infant_s      = scale_to_100(Infant_Mortality)
maternal_s    = scale_to_100(Maternal_Mortality)
physicians_s  = scale_to_100(Physicians_per_1000)
life_s        = scale_to_100(Life_Expectancy)

# Condition matrix and outcome vector
X = hcat(fertility_s, infant_s, maternal_s, physicians_s)
Y = life_s

# Fixed parameters for the consequence (direct method, expert-defined)
α_out_fixed = 0.109861
γ_out_fixed = 70.0
m_out_fixed = 1.0

# Objective function: minimize -(Consistency + Coverage)
# Optimizes only the 12 parameters of the four condition variables
function objective(params)

    α1, γ1, m1 = params[1], params[2], params[3]
    α2, γ2, m2 = params[4], params[5], params[6]
    α3, γ3, m3 = params[7], params[8], params[9]
    α4, γ4, m4 = params[10], params[11], params[12]

    N = length(Y)
    μ_cond = Vector{Float64}(undef, N)
    μ_out  = Vector{Float64}(undef, N)

    for i in 1:N
        μ1 = gclv(X[i,1], α1, γ1, m1)
        μ2 = gclv(X[i,2], α2, γ2, m2)
        μ3 = gclv(X[i,3], α3, γ3, m3)
        μ4 = gclv(X[i,4], α4, γ4, m4)
        μ_cond[i] = min(μ1, μ2, μ3, μ4)
        μ_out[i] = gclv(Y[i], α_out_fixed, γ_out_fixed, m_out_fixed)
    end

    sum_min  = sum(min.(μ_cond, μ_out))
    sum_cond = sum(μ_cond)
    sum_out  = sum(μ_out)

    if sum_cond == 0.0 || sum_out == 0.0
        return Inf
    end

    consistency = sum_min / sum_cond
    coverage    = sum_min / sum_out
    return -(consistency + coverage)
end

# Parameter bounds (12 parameters: 4 variables × 3 params)
lb = [0.05, 0.0, 0.0,  # α, γ, m for variable 1
      0.05, 0.0, 0.0,

      0.05, 0.0, 0.0,
      0.05, 0.0, 0.0]
ub = [3.0, 100.0, 1.0,
      3.0, 100.0, 1.0,
      3.0, 100.0, 1.0,
      3.0, 100.0, 1.0]

search_space = BoxConstrainedSpace(lb=lb, ub=ub)

# Run optimization using ECA (Evolutionary Centers Algorithm)
result = optimize(objective, search_space, ECA(; options=Options(; time_limit=60.0)))

best_params = minimizer(result)
best_value  = -minimum(result)  # since we minimized the negative

# Compute final metrics
function compute_metrics(params)
    α1, γ1, m1 = params[1], params[2], params[3]
    α2, γ2, m2 = params[4], params[5], params[6]
    α3, γ3, m3 = params[7], params[8], params[9]
    α4, γ4, m4 = params[10], params[11], params[12]

    N = length(Y)
    μ_cond = Vector{Float64}(undef, N)
    μ_out  = Vector{Float64}(undef, N)

    for i in 1:N
        μ1 = gclv(X[i,1], α1, γ1, m1)
        μ2 = gclv(X[i,2], α2, γ2, m2)
        μ3 = gclv(X[i,3], α3, γ3, m3)
        μ4 = gclv(X[i,4], α4, γ4, m4)
        μ_cond[i] = min(μ1, μ2, μ3, μ4)
        μ_out[i] = gclv(Y[i], α_out_fixed, γ_out_fixed, m_out_fixed)
    end

    sum_min  = sum(min.(μ_cond, μ_out))
    sum_cond = sum(μ_cond)
    sum_out  = sum(μ_out)

    if sum_cond == 0.0 || sum_out == 0.0
        return (NaN, NaN)
    end
    return (sum_min / sum_cond, sum_min / sum_out)
end

cons, cov = compute_metrics(best_params)
```

The optimized parameters and the resulting consistency and coverage values are reported in Table 8.
